# Evaluating blood–brain barrier permeability in a rat model of type 2 diabetes

**DOI:** 10.1186/s12967-020-02428-3

**Published:** 2020-06-24

**Authors:** Ju Qiao, Christopher M. Lawson, Kilian F. G. Rentrup, Praveen Kulkarni, Craig F. Ferris

**Affiliations:** 1 Center for Translational NeuroImaging, Northeastern University, Boston, MA USA; 2 Department of Psychology, Northeastern University, 360 Huntington Ave, Boston, MA 02115-5000 USA

**Keywords:** Quantitative ultrashort time-to-echo, Contrast enhanced (QUTE-CE), Magnetic resonance imaging, Small vessel disease, BBZDR/Wor rat, Diabetic encephalopathy, Vascular biomarker, Ferumoxytol

## Abstract

**Background:**

This is an exploratory study using a novel imaging modality, quantitative ultrashort time-to-echo, contrast enhanced (QUTE-CE) magnetic resonance imaging to evaluate the permeability of the blood–brain barrier in a rat model of type 2 diabetes with the presumption that small vessel disease is a contributing factor to neuropathology in diabetes.

**Methods:**

The BBZDR/Wor rat, a model of type 2 diabetes, and age-matched controls were studied for changes in blood–brain barrier permeability. QUTE-CE, a quantitative vascular biomarker, generated angiographic images with over 500,000 voxels that were registered to a 3D MRI rat brain atlas providing site-specific information on blood–brain barrier permeability in 173 different brain areas.

**Results:**

In this model of diabetes, without the support of insulin treatment, there was global capillary pathology with over 84% of the brain showing a significant increase in blood–brain barrier permeability over wild-type controls. Areas of the cerebellum and midbrain dopaminergic system were not significantly affected.

**Conclusion:**

Small vessel disease as assessed by permeability in the blood–brain barrier in type 2 diabetes is pervasive and includes much of the brain. The increase in blood–brain barrier permeability is a likely contributing factor to diabetic encephalopathy and dementia.

## Background

Vascular dementia is a serious consequence of diabetes [1]. Prolonged exposure to high blood levels of glucose, typical of type 2 diabetes, affects capillary endothelial structure, function and permeability [2]. Failure in the blood brain barrier lies at the foundation of cerebral small vessel disease and contributes to the pathogenesis of diabetic encephalopathy [3] Methods for in vivo quantification and localization of changes in blood–brain barrier permeability are needed to understand and diagnose the early onset of vascular dementia with type 2 diabetes.

Imaging the subtle changes in blood–brain permeability is not possible with standard imaging protocols but can be assessed with dynamic contrast enhanced (DCE) MRI [4]. However, dynamic contrast enhanced MRI has several limitations. The concentration versus time curve for gadolinium-based contrast agent is typically 15–30% inaccurate; therefore, DCE-MRI has not proven useful clinically [5]. It is also difficult to model the effects of contrast agent on both T2* and T1 given the short acquisition time, and strong dependence on microstructural properties such as vessel size, tortuosity and orientation. These and other methodological issues with the use of DCE-MRI for blood–brain barrier permeability have resulted in significant differences in the reported rates of leakage [5].

To address this issue, a novel imaging modality, quantitative ultrashort time-to-echo, contrast enhanced (QUTE-CE) MRI [6] was used to study changes in blood–brain barrier in the BBZDR/Wor rat an inbred rat strain model of type 2 diabetes [7]. QUTE-CE MRI utilizes Ultrashort-Time-to-Echo (UTE) sequences with ferumoxytol, an FDA-approved superparamagnetic iron oxide nanoparticles (SPIONs) formula already used off-label for human MRI, as a contrast agent to produce positive contrast angiograms with low error of quantification [6, 8].

## Research design and methods

### Animals

This study used male Bio-Breeding Zucker diabetic rats (BBZDR/Wor rats) (n = 8) and age-matched non-diabetic BBDR littermates (n = 7). The founding population was established by Biomere (Worcester, MA). The company decided to retire the breeding line and made a gift of their last animals to the Center for Translational NeuroImaging. The obese male BBZDR/Wor rat spontaneously develops type 2 diabetes at approximately 10 weeks of age (~ 100%) when fed standard rat chow. BBZDR/Wor diabetic rat displays all clinical symptoms typically associated with type 2 diabetes including dyslipidemia, hyperglycemia, insulin resistance, and hypertension [7],

Rats were maintained on a 12 h:12 h light–dark cycle with a light on at 07:00 h, allowed access to food and water ad libitum and were treated with intraperitoneal injections of saline at indications of weight loss. All animal experiments were conducted in accordance with the Northeastern University Division of Laboratory Animal Medicine and Institutional Animal Care and Use Committee. (https://academic.oup.com/ilarjournal/article/45/3/292/704910).

Access to rats was dependent upon the breeding schedule and resulting genotypes. This required we run two separate imaging studies, each with four rats from each genotype, separated by 6 months.

### Imaging

Studies were done on a Bruker Biospec 7.0 T/20 cm USR horizontal magnet (Bruker, Billerica, MA, USA) and a 20-G/cm magnetic field gradient insert (ID = 12 cm) capable of a 120 μs rise time. Radio frequency signals were sent and received with a quadrature volume coil built into the rat restrainer (Animal Imaging Research, Holden, Massachusetts). All rats imaged under 1–2% isoflurane while keeping a respiratory rate of 40–50 breadths/min. At the beginning of each imaging session, a high-resolution anatomical data set was collected using the RARE pulse sequence with following parameters, 35 slice of 0.7 mm thickness; field of view 3 cm; 256 × 256; repetition time [TR] 3900 ms; effective echo time [TE] 48 ms; number of excitations 3; 6 min 14 s acquisition time.

Rats were imaged prior to and following an i.v. bolus of 6 mg/ml Fe of Ferumoxytol. The injected volume was tailored for each rat (assuming 7% blood by body weight) to produce a starting blood concentration of 200 μg/ml Fe (2 × the clinical dose approved for use in humans). The QUTE-CE MRI image parameters of TE = 13 µs, TR = 4 ms, and flip angle = 20° utilized a high radio frequency pulse bandwidth of 200 kHz. Therefore, the pulse duration was short (6.4 µs) compared to the T2 of the approximate ferumoxytol concentration (4.58 ms for 3.58 mM, i.e. 200 µg/ml to minimize signal blur and reduce the probability for a curved trajectory of the magnetization vector Mz. A 3 ×3×3 cm^3^ field-of-view was used with a matrix mesh size of 180× 180×180 to produce 167 µm isotropic resolution.

Images were motion-corrected, aligned spatially, and resliced using MATLAB SPM12 toolbox developed at UCL (https://www.fil.ion.ucl.ac.uk/spm/). The pre-contrast UTE images were set as the baseline. For each rat in each imaging session, the voxel wise percentage change of signal intensity was calculated as (post-con – baseline)/(blood intensity change) *100% as described in our previous work [10], where blood intensity change is a normalization factor calculated by the post-con blood signal intensity minus baseline blood signal intensity. A 173-region rat brain atlas (Ekam Solutions LLC, Boston, MA, US) was fit to T2-weighted RARE anatomical data set for each rat data set taken at each imaging session, using software developed at Northeastern University Center for Translational Neuroimaging (CTNI), considering the variations in brain size and positions. The fitted atlas was transferred to UTE imaging. Once the images were co-registered to the atlas, custom MATLAB code was used to mask individual brain regions for ferumoxytol measurement. Post contrast UTE images are shown for a control and diabetic rat in Additional file 1: Figure S1.

Mode of percentage change distribution for each of the 173 brain areas for control and BBZDR/Wor rats was statistically compared using a Wilcoxon rank-sum test with the alpha set at 0.05. Data was analyzed by co-authors Cai and Kulkarni blind to the identity of the groups.

### Data and resource availability

All data can be accessed through a link to Mendeley. DOI to follow.

## Results

**Fig. 1:**
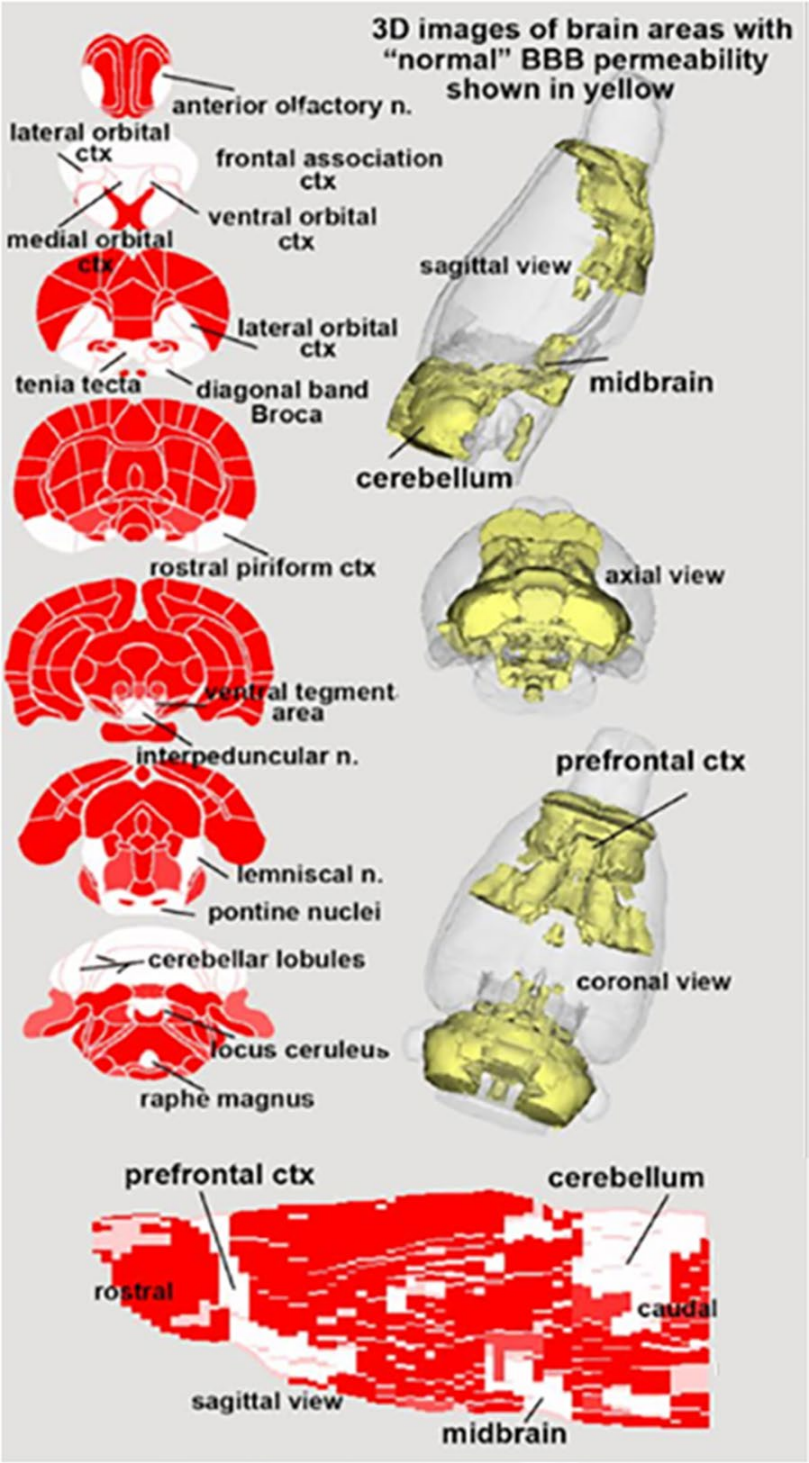
Imaging blood brain barrier permeability. Quantitative ultra-short time-to-echo, contrast enhanced imaging of blood brain barrier permeability comparing BBZDR/Wor rats and their littermate controls. All areas in red show significantly greater permeability in type 2 diabetic as compared to controls

Table 1 shows all the brain areas (147/173) that were significantly different (α p < 0.05) in blood–brain barrier permeability between BBZDR/Wor rats and their littermate controls. Note in all cases BBZDR/Wor rats showed greater permeability. The location of these areas can be are visualized in the surrounding 2D and 3D images generated with the rat MRI atlas shown in Fig. 1. All areas in red in the 2D representations show significantly greater blood–brain barrier permeability in the BBZDR/Wor rats as compared to controls. Table 2 shows all brain areas (26/173) that were not significantly different in blood–brain barrier permeability between BBZDR/Wor rats and their littermate controls. These areas shown in white are localized to the prefrontal ctx, midbrain and cerebellum. These nonaffected areas are coalesced into 3D volumes and pictured in the glass brain in yellow.

**Table 1:** Brain areas that have significantly greater blood–brain barrier permeability in the diabetic BBZDR/Wor rat as compared to wild type controls

Areas with significant changes in blood–brain barrier permeability						
Brain area	Control			Diabetes		*P* value
	Mean	SD		Mean	SD	
Parafascicular thalamic nucleus	0.03	0.00	<	0.08	0.01	0.000
Visual 1 ctx	0.03	0.00	<	0.08	0.01	0.000
Entorhinal ctx	0.03	0.01	<	0.09	0.01	0.000
Dentate gyrus ventral	0.03	0.01	<	0.10	0.01	0.000
Medial geniculate	0.03	0.00	<	0.11	0.01	0.000
Medial dorsal thalamic nucleus	0.02	0.00	<	0.08	0.01	0.000
Visual 2 ctx	0.03	0.00	<	0.09	0.01	0.000
Vuditory ctx	0.03	0.00	<	0.08	0.01	0.000
Ventral posteriolateral thalamic nucleus	0.03	0.00	<	0.07	0.01	0.000
Triangular septal nucleus	0.02	0.01	<	0.07	0.01	0.000
Bed nucleus stria terminalis	0.01	0.00	<	0.05	0.01	0.000
Inferior colliculus	0.04	0.01	<	0.11	0.02	0.000
Posterior thalamic nucleus	0.03	0.00	<	0.07	0.01	0.000
Dorsal lateral striatum	0.02	0.00	<	0.06	0.01	0.000
Lateral posterior thalamic nucleus	0.03	0.01	<	0.09	0.01	0.000
Reticular nucleus	0.03	0.00	<	0.07	0.01	0.000
CA1 dorsal	0.03	0.00	<	0.06	0.01	0.000
Dentate gyrus dorsal	0.03	0.00	<	0.08	0.01	0.000
Central amygdaloid nucleus	0.01	0.01	<	0.05	0.01	0.000
Ventral lateral striatum	0.02	0.00	<	0.06	0.01	0.000
Reuniens nucleus	0.03	0.00	<	0.07	0.01	0.000
Globus pallidus	0.02	0.00	<	0.05	0.01	0.000
Lateral geniculate	0.04	0.01	<	0.09	0.01	0.000
Dorsal medial striatum	0.02	0.00	<	0.06	0.01	0.000
Paraventricular nucleus	0.03	0.00	<	0.07	0.01	0.000
Retrosplenial caudal ctx	0.03	0.01	<	0.12	0.02	0.000
Lateral septal nucleus	0.02	0.00	<	0.06	0.01	0.000
CA2	0.03	0.00	<	0.06	0.01	0.000
Ventrolateral thalamic nucleus	0.03	0.00	<	0.07	0.01	0.000
External plexiform layer	0.07	0.01	<	0.12	0.01	0.000
Periaqueductal gray thalamus	0.04	0.00	<	0.08	0.01	0.000
Temporal ctx	0.03	0.01	<	0.12	0.02	0.000
Ventral subiculum	0.04	0.00	<	0.08	0.01	0.000
Ventral posteriomedial thalamic nucleus	0.03	0.00	<	0.08	0.01	0.000
Basal amygdaloid nucleus	0.01	0.01	<	0.06	0.01	0.000
Ventromedial thalamic nucleus	0.03	0.00	<	0.08	0.01	0.000
Parietal ctx	0.03	0.00	<	0.06	0.01	0.000
Caudal piriform ctx	0.03	0.00	<	0.08	0.02	0.000
Medial amygdaloid nucleus	0.03	0.01	<	0.09	0.02	0.000
CA1 hippocampus ventral	0.04	0.01	<	0.08	0.01	0.000
Primary somatosensory ctx barrel field	0.03	0.00	<	0.07	0.01	0.000
Zona incerta	0.04	0.01	<	0.08	0.01	0.000
Primary somatosensory ctx forelimb	0.02	0.00	<	0.06	0.01	0.000
Granular cell layer	0.06	0.01	<	0.10	0.01	0.000
Habenula nucleus	0.06	0.01	<	0.15	0.03	0.000
Primary somatosensory ctx trunk	0.03	0.00	<	0.06	0.01	0.000
Lateral hypothalamus	0.04	0.00	<	0.08	0.02	0.000
Primary somatosensory ctx shoulder	0.02	0.01	<	0.06	0.01	0.000
Ventral medial striatum	0.02	0.00	<	0.05	0.01	0.000
Glomerular layer	0.09	0.01	<	0.14	0.02	0.000
Prerubral field	0.04	0.01	<	0.08	0.01	0.000
Extended amygdala	0.02	0.00	<	0.05	0.01	0.000
Anterior hypothalamic area	0.02	0.00	<	0.06	0.01	0.000
Primary motor ctx	0.02	0.00	<	0.06	0.01	0.000
Secondary somatosensory ctx	0.03	0.00	<	0.07	0.01	0.000
Intercalated amygdaloid nucleus	0.01	0.01	<	0.07	0.01	0.000
Primary somatosensory ctx upper lip	0.03	0.00	<	0.07	0.01	0.000
White matter rostral	0.03	0.00	<	0.06	0.01	0.000
CA3 dorsal	0.03	0.00	<	0.06	0.01	0.000
Posterior hypothalamic area	0.04	0.01	<	0.10	0.02	0.000
Central medial thalamic nucleus	0.04	0.00	<	0.07	0.01	0.000
Dorsal raphe	0.04	0.01	<	0.08	0.01	0.000
Supramammillary nucleus	0.05	0.01	<	0.15	0.04	0.000
Primary somatosensory ctx hindlimb	0.03	0.00	<	0.06	0.01	0.000
Ventral anterior thalamic nucleus	0.03	0.01	<	0.07	0.01	0.000
Lateral amygdaloid nucleus	0.03	0.01	<	0.07	0.01	0.000
Claustrum	0.02	0.00	<	0.05	0.01	0.000
Perirhinal ctx	0.05	0.01	<	0.12	0.02	0.000
Lateral dorsal thalamic nucleus	0.03	0.01	<	0.06	0.01	0.000
Dorsal medial nucleus	0.03	0.01	<	0.07	0.01	0.000
Ectorhinal ctx	0.04	0.01	<	0.15	0.05	0.000
Olivary nucleus	0.05	0.01	<	0.09	0.02	0.000
Copula of the pyramis	0.07	0.01	<	0.11	0.02	0.000
Motor trigeminal nucleus	0.04	0.01	<	0.07	0.01	0.000
Paramedian lobule	0.06	0.00	<	0.08	0.01	0.000
Solitary tract nucleus	0.03	0.01	<	0.06	0.01	0.000
Parvicellular reticular areas	0.04	0.00	<	0.06	0.01	0.000
Precuniform nucleus	0.04	0.01	<	0.07	0.01	0.000
Anterior cingulate area	0.03	0.00	<	0.08	0.02	0.000
Cortical amygdaloid nucleus	0.05	0.01	<	0.10	0.02	0.000
Primary somatosensory ctx jaw	0.03	0.01	<	0.06	0.01	0.000
Parabrachial nucleus	0.05	0.01	<	0.08	0.01	0.000
Principal sensory nucleus trigeminal	0.05	0.00	<	0.07	0.01	0.000
Sub coeruleus nucleus	0.04	0.00	<	0.06	0.01	0.000
White matter caudal	0.04	0.00	<	0.07	0.02	0.000
Endopiriform nucleus	0.02	0.01	<	0.05	0.01	0.000
Reticular nucleus midbrain	0.04	0.01	<	0.08	0.02	0.000
Anterior thalamic nuclei	0.03	0.01	<	0.07	0.02	0.000
Accumbens core	0.02	0.01	<	0.05	0.02	0.000
Prelimbic ctx	0.03	0.00	<	0.06	0.02	0.000
7th cerebellar lobule	0.03	0.01	<	0.06	0.01	0.000
CA3 hippocampus ventral	0.04	0.01	<	0.08	0.02	0.000
Ventral medial nucleus	0.03	0.01	<	0.08	0.03	0.000
Dorsal paragigantocellularis	0.03	0.01	<	0.05	0.01	0.000
Median raphe nucleus	0.04	0.01	<	0.06	0.01	0.000
Pedunculopontine tegmental area	0.04	0.01	<	0.07	0.02	0.000
Secondary motor ctx	0.03	0.01	<	0.07	0.02	0.000
Central gray	0.05	0.00	<	0.08	0.01	0.000
Retrosplenial rostral ctx	0.05	0.01	<	0.11	0.03	0.001
Subthalamic nucleus	0.07	0.01	<	0.11	0.02	0.001
Medial preoptic area	0.02	0.01	<	0.05	0.01	0.001
Medial septum	0.03	0.01	<	0.06	0.01	0.001
Gigantocellularis reticular nucleus pons	0.03	0.00	<	0.05	0.01	0.001
Superior colliculus	0.04	0.01	<	0.07	0.02	0.001
Subiculum dorsal	0.04	0.00	<	0.06	0.01	0.001
Lateral preoptic area	0.02	0.01	<	0.05	0.02	0.001
Magnocellular preoptic nucleus	0.03	0.01	<	0.08	0.03	0.001
Dorsomedial tegmental area	0.04	0.01	<	0.06	0.02	0.001
Neural lobe pituitary	0.14	0.05	<	0.26	0.06	0.001
Medial cerebellar nucleus fastigial	0.06	0.01	<	0.08	0.01	0.001
Substantia nigra compacta	0.05	0.01	<	0.10	0.03	0.001
8th cerebellar lobule	0.04	0.01	<	0.06	0.01	0.001
Medial mammillary nucleus	0.07	0.04	<	0.20	0.08	0.002
Pontine reticular nucleus caudal	0.03	0.00	<	0.05	0.01	0.002
Flocculus cerebellum	0.05	0.01	<	0.08	0.02	0.002
Substantia nigra reticularis	0.07	0.02	<	0.12	0.03	0.002
Supraoptic nucleus	0.05	0.01	<	0.08	0.02	0.002
Reticulotegmental nucleus	0.03	0.01	<	0.05	0.01	0.003
Anterior lobe pituitary	0.17	0.02	<	0.28	0.07	0.003
Accumbens shell	0.03	0.01	<	0.06	0.02	0.003
Inferior olivary complex	0.05	0.00	<	0.07	0.02	0.003
10th cerebellar lobule	0.07	0.01	<	0.09	0.01	0.003
Infralimbic ctx	0.03	0.01	<	0.07	0.02	0.003
Cochlear nucleus	0.06	0.01	<	0.08	0.02	0.004
Premammillary nucleus	0.05	0.02	<	0.11	0.04	0.004
Insular ctx	0.04	0.01	<	0.07	0.02	0.004
Red nucleus	0.05	0.01	<	0.08	0.02	0.004
Suprachiasmatic nucleus	0.01	0.02	<	0.05	0.02	0.005
Root of trigeminal nerve	0.05	0.00	<	0.07	0.02	0.005
Interposed nucleus	0.06	0.01	<	0.08	0.01	0.006
Vestibular nucleus	0.05	0.01	<	0.06	0.01	0.006
9th cerebellar lobule	0.05	0.01	<	0.07	0.01	0.007
2nd cerebellar lobule	0.07	0.01	<	0.10	0.02	0.007
Pontine reticular nucleus oral	0.04	0.01	<	0.05	0.01	0.008
Retrochiasmatic nucleus	0.05	0.04	<	0.13	0.06	0.009
Anterior pretectal nucleus	0.03	0.00	<	0.07	0.03	0.009
Trapezoid body	0.03	0.01	<	0.06	0.02	0.010
Facial nucleus	0.05	0.01	<	0.07	0.02	0.010
Raphe obscurus nucleus	0.03	0.01	<	0.04	0.01	0.011
Ventral pallidum	0.04	0.01	<	0.07	0.02	0.011
Raphe linear	0.06	0.01	<	0.08	0.02	0.012
Periolivary nucleus	0.05	0.02	<	0.08	0.03	0.019
Dentate n. cerebellum	0.05	0.01	<	0.06	0.02	0.019
Arcuate nucleus	0.07	0.04	<	0.13	0.06	0.023
Substantia innominata	0.05	0.02	<	0.09	0.03	0.024
Paraflocculus cerebellum	0.06	0.01	<	0.07	0.02	0.026
Anterior amygdaloid nucleus	0.03	0.01	<	0.05	0.03	0.035

Areas are ranked in order of their significance (α < 0.05). False detection rate (α = 0.17)

**Table 2:** Brain areas that show no significant differences in blood–brain barrier permeability in the diabetic BBZDR/Wor rat as compared to wild type controls

Areas with nonsignificant changes in blood–brain barrier permeability						
Brain area	Control			Diabetes		
	Mean	SD		Mean	SD	*P *value
Olfactory tubercles	0.07	0.02	<	0.10	0.04	0.067
Medial pretectal area	0.00	0.16	<	0.17	0.18	0.070
Raphe magnus	0.03	0.01	<	0.05	0.02	0.088
Paraventricular nucleus	0.06	0.02	<	0.09	0.05	0.091
3rd cerebellar lobule	0.07	0.01	<	0.09	0.03	0.110
Ventral tegmental area	0.07	0.02	<	0.11	0.06	0.130
Rostral piriform ctx	0.05	0.02	<	0.07	0.03	0.143
Locus coeruleus	0.07	0.01	<	0.08	0.01	0.146
Diagonal band of Broca	0.04	0.02	<	0.06	0.03	0.157
Ventral orbital ctx	0.04	0.01	<	0.06	0.03	0.166
6th cerebellar lobule	0.04	0.01	<	0.05	0.01	0.200
Lateral orbital ctx	0.04	0.01	<	0.06	0.03	0.285
Anterior olfactory nucleus	0.05	0.01	<	0.07	0.04	0.289
Tenia tecta ctx	0.06	0.02	<	0.09	0.06	0.290
1st cerebellar lobule	0.05	0.01	<	0.06	0.02	0.353
Simple lobule cerebellum	0.09	0.01	>	0.07	0.05	0.423
Pontine nuclei	0.05	0.03	<	0.07	0.05	0.451
Pineal gland	0.86	0.04	<	0.90	0.12	0.458
Interpeduncular nucleus	0.09	0.04	<	0.11	0.08	0.522
Medial orbital ctx	0.09	0.01	>	0.08	0.05	0.526
Crus 2 of ansiform lobule	0.06	0.00		0.06	0.01	0.604
Lemniscal nucleus	0.06	0.02	<	0.07	0.03	0.611
Frontal association ctx	0.08	0.01		0.08	0.04	0.658
Crus 1 of ansiform lobule	0.06	0.01	>	0.05	0.02	0.790
4th cerebellar lobule	0.07	0.01		0.07	0.04	0.809
5th cerebellar lobule	0.07	0.01	>	0.06	0.04	0.856

Areas are ranked in order of their *P* values

## Discussion

QUTE-CE MRI, was developed as a quantitative vascular biomarker [6]. Ferumoxytol (Feraheme™) MRI with optimized 3D Ultra-Short Time-to-echo (UTE) Pulse Sequences produces angiographic images unparalleled to time-of-flight imaging or gadolinium-based first-pass imaging. The contrast agent is ferumoxytol, an ultra-small superparamagnetic iron oxide nanoparticle with a dextran coating. Since the size exceeds the cutoff (~ 6 nm) for glomerular filtration, ferumoxytol is not cleared by the kidney, and instead is an excellent blood pool contrast agent with a long intravascular half-life of ~ 15 h [9]**.** Numerous clinical MRI studies using ferumoxytol have been conducted in children and adults, demonstrating no major adverse effects, thus QUTE-CE can be readily used in the clinic to study blood–brain barrier permeability [10]. We recently published a study mapping the absolute physiological cerebral blood volume (CBV) of the awake rat brain, including measurements of microvasculature density and vascular functional reserve [8]. QUTE-CE MRI can be used for identifying hyper- or hypo-vascularization, small vessel density, blood–brain barrier permeability and vascular reserve and vascular responsivity to CO2 challenge at the individual voxel and regional levels using our rat 3D MRI atlas. As demonstrated in this study with the BBZDR/Wor rats, a preclinical model of type 2 diabetes, this imaging technology could be used to diagnose and evaluate blood brain permeability and disease progression in diabetic encephalopathy in the clinic.

## Limitations and future directions

As a pilot study with a small population of rats there were several limitations: (1) Females were not studied. Unfortunately, only males develop diabetes in the BBZDR/Wor strain of rats [7]. (2) While the blood–brain permeability was pervasive in this late-stage model of diabetes and not unexpected, postmortem histology would have confirmed the capillary pathology and helped to understand why areas like the cerebellum and midbrain were spared. (3) In the future, a thorough comparison between DCE and QUTE-CE should be done to provide quantitative data on the differences and similarities between both imaging techniques. (4) More common rat models of T2DB should be tested like the Goto-Kakizaki GK rat [11] or high-fat diet, streptozotocin treated Wistar rat (HFD/STZ) [12].

## Conclusion

Small vessel disease as assessed by permeability in the blood–brain barrier in type 2 diabetes is pervasive and includes much of the brain. The increase in blood–brain barrier permeability is a likely contributing factor to diabetic encephalopathy and dementia.

## Supplementary information

**Additional file 1: Figure S1.** Shown are raw data from a control and diabetic rat following ferumoxytol injection. The normalized UTE signal is registered to the original anatomy.

## Data Availability

All data can be accessed through a link to Mendeley. DOI to follow.

## Abbreviations

QUTE-CE: Quantitative ultrashort time-to-echo, contrast enhanced
BBB: Blood brain barrier
CBV: Cerebral blood volume
DCE: Dynamic contrast enhanced
BBZDR: Male Bio-Breeding Zucker diabetic rats
TR: Repetition time
TE: Echo time
RARE: Rapid acquisition with relaxation enhancement
