# Supplementary material for: Evaluating blood–brain barrier permeability in a rat model of type 2 diabetes
Source: J Transl Med. 2020 Jun 24;18:256. doi: 10.1186/s12967-020-02428-3 (PMC7313122; doi:10.1186/s12967-020-02428-3)
Supplement: Supplementary file 1 — Additional file 1: Figure S1. Shown are raw data from a control and diabetic rat following ferumoxytol injection. The normalized UTE signal is registered to the original anatomy. [file 12967_2020_2428_MOESM1_ESM.docx]

**
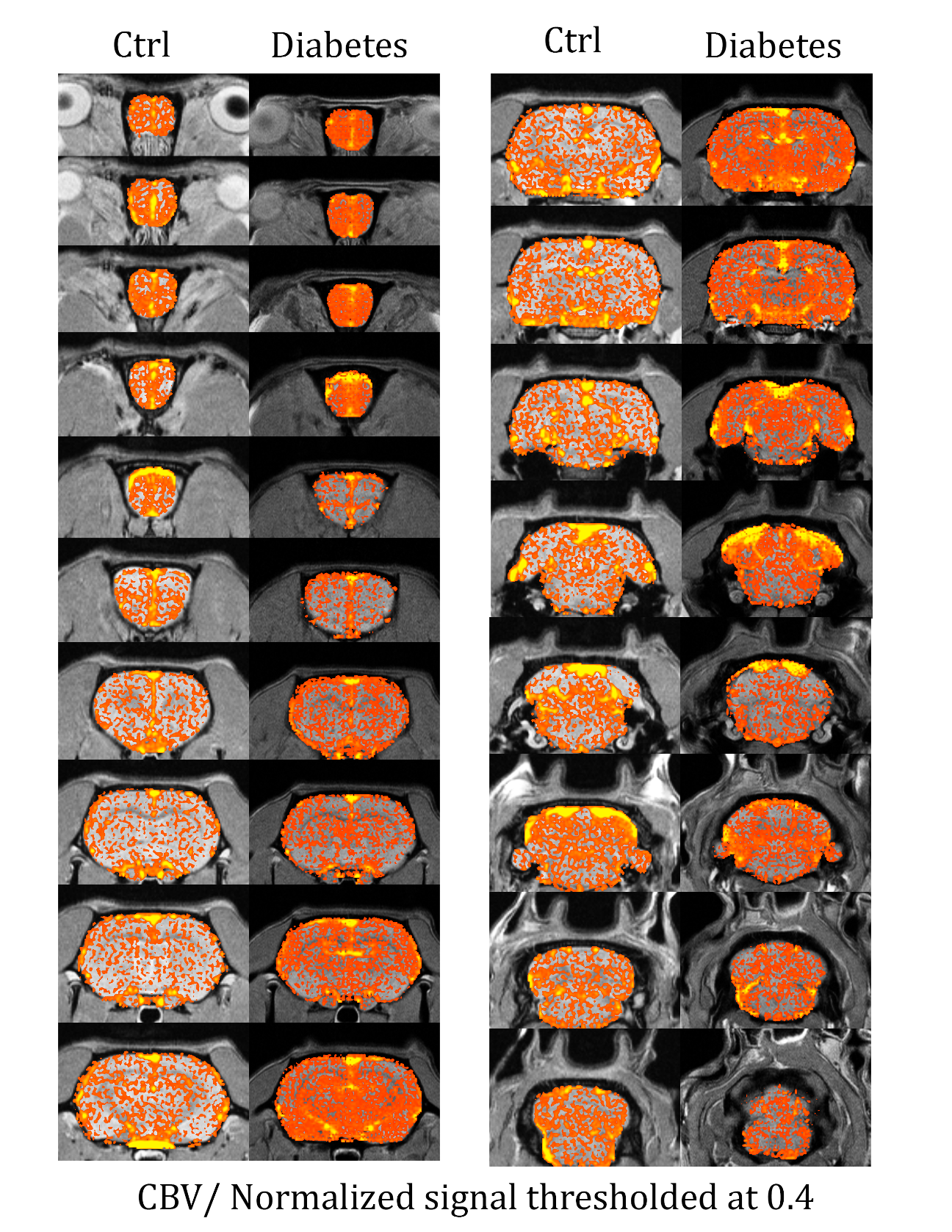
Figure S1** Shown are raw data from a Control and Diabetic rat following ferumoxytol injection. The normalized UTE signal is registered to the original anatomy.
